# Jarzyski’s Equality and Crooks’ Fluctuation Theorem for General Markov Chains with Application to Decision-Making Systems

**DOI:** 10.3390/e24121731

**Published:** 2022-11-27

**Authors:** Pedro Hack, Sebastian Gottwald, Daniel A. Braun

**Affiliations:** Institute of Neural Information Processing, Ulm University, 89081 Ulm, Germany

**Keywords:** fluctuation theorems, Markov chains, information processing, decision-making

## Abstract

We define common thermodynamic concepts purely within the framework of general Markov chains and derive Jarzynski’s equality and Crooks’ fluctuation theorem in this setup. In particular, we regard the discrete-time case, which leads to an asymmetry in the definition of work that appears in the usual formulation of Crooks’ fluctuation theorem. We show how this asymmetry can be avoided with an additional condition regarding the energy protocol. The general formulation in terms of Markov chains allows transferring the results to other application areas outside of physics. Here, we discuss how this framework can be applied in the context of decision-making. This involves the definition of the relevant quantities, the assumptions that need to be made for the different fluctuation theorems to hold, as well as the consideration of discrete trajectories instead of the continuous trajectories, which are relevant in physics.

## 1. Introduction

Over the last 20 years, several advances in thermodynamics have led to the development of results relating equilibrium quantities to nonequilibrium trajectories. Those advances have crystallized in a new area of research, *nonequilibrium thermodynamics*, where these relations play a major role [1,2,3]. Among them, two of the most remarkable are Jarzynski’s equality [4,5,6] and Crooks’ fluctuation theorem [7,8], for which experimental evidence has been reported in several contexts: unfolding and refolding processes involving RNA [9,10], electronic transitions between electrodes manipulating a charge parameter [11], the rotation of a macroscopic object inside a fluid surrounded by magnets where the current of a wire attached to the macroscopic object is manipulated [12], and a trapped-ion system [13,14].

**Figure 1:**
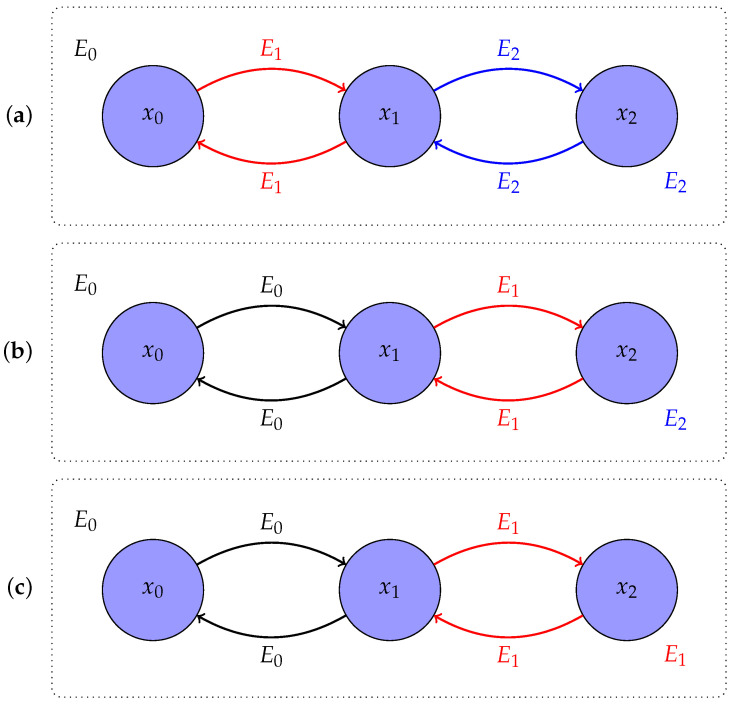
Relation between a forward process, its corresponding backward process, and the definition of work. We consider a trajectory $x=(x_{0},x_{1},x_{2})$ and three energy functions $E_{0},E_{1},E_{2}$. The upper (bottom) line of arrows represents the forward (backward) process. A work step $W_{i}=E_{i}\left(x_{i-1}\right)-E_{i-1}\left(x_{i-1}\right)$ is typically defined as the change in energy due to the external change of the energy function, whereas a heat step $Q_{i}=E_{i}\left(x_{i}\right)-E_{i}\left(x_{i-1}\right)$ is defined as the change in energy due to internal state changes. (**a**) Typical relation between the forward and backward processes in physics [8]. Work in the forward process would be $W_{F}=E_{1}\left(x_{0}\right)-E_{0}\left(x_{0}\right)+E_{2}\left(x_{1}\right)-E_{1}\left(x_{1}\right)$, whereas the backward work under the same definition would be $W_{B}=E_{1}\left(x_{1}\right)-E_{2}\left(x_{1}\right)$. Instead, backward work is usually defined as $W_{B}=E_{1}\left(x_{1}\right)-E_{2}\left(x_{1}\right)+E_{0}\left(x_{0}\right)-E_{1}\left(x_{0}\right)$ to fulfil the physical time reversal symmetry $W_{F}=-W_{B}$. (**b**) Another typical protocol in physics [15,17]. In this case, the asymmetry is the other way round, where $E_{2}$ does not influence the forward process. (**c**) Symmetric protocol where both forward and backward work follow the same definition with $W_{F}=E_{1}\left(x_{1}\right)-E_{0}\left(x_{0}\right)$ and $W_{B}=E_{0}\left(x_{1}\right)-E_{1}\left(x_{1}\right)=-W_{F}$. This is the protocol we propose in Section 4.

These two results have been derived under several assumptions in the context of nonequilibrium thermodynamics, including both deterministic [5,6] and stochastic dynamics [4,7,8,15]. Moreover, it has been argued that both results can be obtained as a consequence of Bayesian retrodiction in a physical context [16]. Here, we derive both of them using only the concepts from the theory of Markov chains. This allows us to both distinguish the mathematical from the physical assumptions underlying them and, thus, to make them available for application in other areas where the framework of thermodynamics may be useful. The distinction between mathematical and physical assumptions will be of particular importance for the definition of work, as we will see, since the usual definition based on physical considerations leads to an asymmetry of the definition in processes that run either forward or backward in time—see Figure 1 for a simple example. This is relevant, for instance, when analysing trajectories in terms of their work value, if we do not know whether they were recorded in the forward direction or whether they have been generated by playing them backwards. Ideally, we would like to be able to ascribe work values directly to trajectories without any additional information.

One of the application areas where the framework of thermodynamics has recently been investigated outside the realm of physics is the analysis of simple learning systems [1,18,19,20,21,22,23,24,25,26] and, in particular, the problem of decision-making under uncertainty and resource constraints [22,27,28,29,30,31]. The basic analogy follows from the idea that decision-making involves two opposing *forces*: (i) the tendency of the decision-maker toward better options (equivalently, to maximize a function called *utility*) and (ii) the restrictions on this tendency given by the limited information-processing capabilities of the decision-maker, which prevents him/her from always picking the best option and is usually modelled by a bound on the entropy of the probability distribution that describes the decision-maker’s behaviour. Thermodynamic systems are also explained in terms of two opposing forces, the first being the energy, which the system tries to minimize, and the second being the entropy, which prevents the minimization of the energy to its full extent. Thus, in both cases, we formally deal with optimization problems under information constraints, and thus, we can conceptualize both decision-making and thermodynamics in terms of information theory. In particular, we can consider the environment in which a decision is being made or a thermodynamic system is immersed as a source of information (in the form of either utility or energy), which, due to the noise (modelled by entropy), reaches the decision-making or thermodynamic system with some error. This results in an imperfect response by the system.

The analogy between thermodynamics and decision-making is not restricted to the equilibrium case [22,27,28,29,30,31], but can be taken further from equilibrium to nonequilibrium systems. In particular, the aforementioned fluctuation theorems of Jarzynski and Crooks have been previously suggested to apply to decision-makers that adapt to changing environments [32]. In this previous work, hysteresis and adaptation were investigated in decision-makers, however, based on the physical convention of defining work differently for forward and backward processes. Here, we improve on the work there by replacing this convention with a different energy protocol that naturally entails a symmetric definition of work and by weakening the assumptions that are actually needed in order for the fluctuation theorems to hold in the context of general Markov chains. Given the fact that the literature on this topic belongs for the most part to thermodynamics, we adopt the thermodynamic notation here. In particular, we consider energy functions instead of utilities and take Markov chains as the starting point.

Our manuscript is organized as follows. In Section 2, we introduce the notions of work and other thermodynamic concepts that are inherent to Markov chains, that is, in contrast to the formalism in physics [7,8,15], we start with the assumption of a Markov chain and deduce all other concepts from that without the need to presuppose the existence of an external energy function. We discuss under what conditions these concepts are uniquely specified from the Markov chain. In Section 3, we use this framework to weaken the derivation of Jarzynski’s equality (Theorem 1) in the context of decision-making that was presented in [32]. In Section 4, we prove Crooks’ fluctuation theorem (Theorem 2 and Corollary 2) within the same setup. In particular, we use an additional assumption that is not mandatory in Crooks’ work [7,8,15], but here is needed given the inherent nature of our definition of work. In fact, we provide an example in which the new requirement is violated and, as a consequence, Crooks’ theorem is false everywhere (Proposition 3). In Section 5, we discuss how the concepts we have developed can be applied to decision-making systems.

Notice, for simplicity, we develop the results for discrete-time Markov chains with finite state spaces. However, the ideas can be translated, for example, to continuous state spaces by assuming for densities of Markov kernels the properties we use here for transition matrices. We include a few more details regarding this scenario in the discussion (Section 5), where we also briefly address the case of continuous-time Markov chains.

## 2. Thermodynamics for Markov Chains

### 2.1. Definitions of Energy, Heat, and Work

In this section, we assume there is some stochastic process that can be modelled as a Markov chain and discuss the definition of the energy, partition function, free energy, work, heat, and dissipated work in such a context. We follow the terminology in [33].

We call a finite number of random variables $X={\left(X_{n}\right)}_{n=0}^{N}$ over a finite state space *S* a *Markov chain* if we have for any 0<n≤N that

$$P\left(X_{n}=x_{n}|X_{0}=x_{0},\ldots ,X_{n-1}=x_{n-1}\right)≔P\left(X_{n}=x_{n}|X_{n-1}=x_{n-1}\right)$$

for all $\left(x_{0},x_{1},\ldots ,x_{n}\right)\in S^{n+1}$. Notice that we can characterize X by a probability distribution $p_{0}$, where $p_{0}\left(x\right)=P\left(X_{0}=x\right)$ and *N transition matrices* ${\left(M_{n}\right)}_{n=1}^{N}$ given by

$${\left(M_{n}\right)}_{xy}≔P\left(X_{n}=x|X_{n-1}=y\right)$$

for all x,y∈S and 1≤n≤N. Notice that any transition matrix $M_{n}$ is a *stochastic matrix*, that is we have $\sum_{x\in S}{\left(M_{n}\right)}_{xy}=1$ for all y∈S.

If *p* is a distribution, $M_{n}$ a transition matrix of X for some fixed *n*, and we have $M_{n}p=p$, where $\left(M_{n}p\right)\left(x\right)≔\sum_{y\in S}{\left(M_{n}\right)}_{xy}p\left(y\right)$, then we say *p* is a *stationary distribution* of $M_{n}$. Note that this terminology applies to a *single* $M_{n}$, that is a stationary distribution *p* of $M_{n}$ is stationary with respect to the (homogeneous) Markovian dynamics of that fixed transition matrix, and generally not with respect to the (inhomogeneous) Markovian dynamics of X. If *p* fulfils

(1) $${\left(M_{n}\right)}_{yx}p\left(x\right)={\left(M_{n}\right)}_{xy}p\left(y\right)\;\forall x,y\in S,$$

then we say *p* satisfies *detailed balance* with respect to $M_{n}$. Note that such a *p* is a stationary distribution of $M_{n}$. In case $M_{n}$ has a unique stationary distribution *p* that satisfies detailed balance with respect to $M_{n}$, then we may simply say $M_{n}$ satisfies detailed balance. We say a transition matrix $M_{n}$ is *irreducible* if, for any pair x,y∈S, there exists an integer m≥1 such that ${\left(M_{n}^{m}\right)}_{xy}>0$. Irreducible transition matrices have a useful property, which we present in Lemma 1 (see ([33], Corollary 1.17 and Proposition 1.19) for a proof).

**Lemma 1.**  *If a transition matrix is irreducible, then it has a unique stationary distribution. Furthermore, the stationary distribution has non-zero entries.*

If X has initial distribution $p_{0}$, irreducible transition matrices ${\left(M_{n}\right)}_{n=1}^{N}$, and $p_{N}$ is the unique stationary distribution of $M_{N}$, then we say the Markov chain $Y≔{\left(Y_{n}\right)}_{n=0}^{N}$ with initial distribution $p_{N}$ and transition matrices ${\left(M_{N-(n-1)}\right)}_{n=1}^{N}$ is the *time reversal* of X. Notice that there are different notions of time reversal. A discussion can be found in ([34], Section III).

Given a distribution *p* on *S* such that p(x)>0 for all x∈S and some β>0, we can associate with *p* an *energy function E*, that is a function E:S→R such that

$$p\left(x\right)=\frac{1}{Z}e^{-\beta E(x)}\;\forall x\in S\;,$$

where $Z≔\sum_{x\in S}e^{-\beta E(x)}$ is called a *partition function* and $F≔-\frac{1}{\beta }\log\left(Z\right)$ the corresponding *free energy*. Notice that, given a distribution *p* with two energy functions *E* and $E^{\prime }$, there is a constant c∈R such that we have

(2) $$E\left(x\right)=E^{\prime }\left(x\right)+c\;\forall x\in S$$

and, accordingly,

(3) $$F=F^{\prime }+c$$

where *F* and $F^{\prime }$ are the free energies for *p* using *E* and $E^{\prime }$, respectively. Thus, each distribution where all entries are strictly positive has, up to a constant, a unique energy function associated with it.

For the remainder of this section, let $X≔{\left(X_{n}\right)}_{n=0}^{N}$ be a Markov chain such that $p_{0}$ has non-zero entries and each transition matrix $M_{n}$ has a unique stationary distribution $p_{n}$ with non-zero entries. In this context, we call a family $E={\left(E_{n}\right)}_{n=0}^{N}$ of functions $E_{n}:S\to R$ a *family of energies* of X, if $E_{n}$ is an energy function of $p_{n}$ for all 0≤n≤N. We define the *work* of a realization $x=\left(x_{0},x_{1},..,x_{N}\right)\in S^{N+1}$ of X, with respect to a family of energies E of X, as

(4) $$W_{X,E}\left(x\right)≔\sum_{n=0}^{N-1}E_{n+1}\left(x_{n}\right)-E_{n}\left(x_{n}\right)\;.$$

Given another family of energies $E^{\prime }={\left(E_{n}^{\prime }\right)}_{n=0}^{N}$ of X, we have

(5) $$W_{X,E}\left(x\right)=W_{X,E^{\prime }}\left(x\right)+\left(c_{N}-c_{0}\right)\;,$$

where $c_{n}≔E_{n}-E_{n}^{\prime }$ are constants by (2). Hence, without fixing a family of energies of X, the work defined in (4) is unique up to a constant. Whenever X and E are clear from the context, we may simply use *W* instead of $W_{X,E}$ for brevity.

Similarly, the *heat* of a realization x of X with respect to a family of energies E is given by

(6) $$Q_{X,E}\left(x\right)≔\sum_{n=1}^{N}E_{n}\left(x_{n}\right)-E_{n}\left(x_{n-1}\right)\;.$$

Given another family of energies $E^{\prime }≔{\left(E_{n}^{\prime }\right)}_{n=0}^{N}$ of X, we have

(7) $$Q_{X,E}\left(x\right)=Q_{X,E^{\prime }}\left(x\right)$$

by (2). We may, thus, use $Q_{X}$ instead of $Q_{X,E}$, or even *Q* in case X is clear.

Moreover, if $F_{n}$ is the free energy associated with $p_{n}$ for any 0≤n≤N, we call $\Delta F_{X,E}≔F_{N}-F_{0}$ the *free energy difference* associated with E, satisfying

(8) $$\Delta F_{X,E}=\Delta F_{X,E^{\prime }}+\left(c_{N}-c_{0}\right)$$

by (3). While both $W_{X,E}$ and $\Delta F_{X,E}$ depend on the difference between the constants $c_{N}$ and $c_{0}$, the so-called *dissipated work*:

$$W_{X,E}^{d}\left(x\right)≔W_{X,E}\left(x\right)-\Delta F_{X,E}$$

does not, that is, for any realization x of X, we have

(9) $$W_{X,E}^{d}\left(x\right)=W_{X,E^{\prime }}^{d}\left(x\right)$$

as a consequence of (5) and (8).

Notice, in the context of the Markov chain framework we have adopted here, the first law of thermodynamics is a direct consequence of the definitions of work and heat (see (12) below):

$$W_{X,E}\left(x\right)+Q_{X,E}\left(x\right)=E_{N}\left(x_{N}\right)-E_{0}\left(x_{0}\right).$$

The second law of thermodynamics can be obtained as well, which is a direct consequence of Jarzynski’s equality, as we will see in Section 5.

### 2.2. Main Result: Fluctuation Theorems for Markov Chains

Our main results are versions of *Jarzynski’s equality* [4,5,6] and *Crooks’ fluctuation theorem* [7,8,15] for Markov chains, the derivations of which can be found in Section 3 and Section 4 below.

Consider a Markov chain $X={\left(X_{n}\right)}_{n=0}^{N}$ on a finite state space whose initial distribution $p_{0}$ has non-zero entries and whose transition matrices ${\left(M_{n}\right)}_{n=1}^{N}$ are irreducible. Then, for any family of energies $E={\left(E_{n}\right)}_{n=0}^{N}$ of X, we have

$$\langle e^{-\beta (W(X)-\Delta F)}\rangle =1\;,$$

where 〈·〉 denotes the expectation operator and $W=W_{X,E}$, $\Delta F=\Delta F_{X,E}$. In physics, this result is known as Jarzynski’s equality, which has been shown in the past to hold under various conditions. In Section 3, we give a simple proof in the context of Markov chains as a direct consequence of the definitions of work and free energy (Theorem 1).

Moreover, if all transition matrices of X satisfy detailed balance and $p_{0}$ is the stationary distribution of $M_{1}$, then for any possible work value, *w*, we have

$$\frac{P(W_{X,E}=w)}{P(W_{Y,\hat{E}}=-w)}=e^{\beta (w-\Delta F)},$$

where Y is the time reversal of X with energies $\hat{E}={\left({\hat{E}}_{n}\right)}_{n=0}^{N}$. The analogous result in physics is known as Crooks’ fluctuation theorem. In Section 4, we prove a slightly more general version in the context of Markov chains without detailed balance (Theorem 2), which is then applied in Corollary 2 to obtain Crooks’ theorem.

## 3. Jarzynski’s Equality for Markov Chains

Jarzynski’s equation was originally derived for deterministic dynamics [5,6] (see also [35]) and later extended to stochastic dynamics [4] using a Master equation approach. Shortly after that, it was shown in the non-deterministic Markov chain context relying on assumptions about the time reversal of the dynamics [8]. In Theorem 1 below, we see that, in the context of Markov chains, Jarzynski’s equality is a straightforward consequence of the definitions of work and free energy. Importantly, it does not require any assumptions regarding time reversal, in contrast to the requirements in a previous decision-theoretic approach to fluctuation theorems in [32].

For the proof of Jarzynski’s equality for Markov chains, the basic observation is that we start with the expected value of a quantity closely related to the equilibrium distributions of our initial Markov chain X, namely $e^{-\beta W(X)}$. With this in mind, we define a new Markov chain Y using the transition matrices of X and the equilibrium distributions of the individual steps. In particular, we define it in a way such that we cancel the dependency of $e^{-\beta W(X)}$ on X and end up with a constant, whose expected value over Y is the constant itself. We include the details in the following theorem.

**Theorem 1** (Jarzynski’s equality for Markov chains)**.**  *If $X={\left(X_{n}\right)}_{n=0}^{N}$ is a Markov chain on a finite state space S whose initial distribution $p_{0}$ has non-zero entries and whose transition matrices ${\left(M_{n}\right)}_{n=1}^{N}$ are irreducible, then we have, for any family of energies $E={\left(E_{n}\right)}_{n=0}^{N}$ of X,*

(10) $$\langle e^{-\beta (W(X)-\Delta F)}\rangle =1\;,$$

*where $W=W_{X,E}$ and $\Delta F=\Delta F_{X,E}$.*

**Proof.**  Notice that, by Lemma 1, we have $p_{n}\left(x\right)>0$ for all x∈S and 0≤n≤N. We first define a new Markov chain $Y≔{\left(Y_{n}\right)}_{n=0}^{N}$ with initial distribution $p_{N}$ and with transition matrices ${\left({\hat{M}}_{n}\right)}_{n=1}^{N}$, where for all x,y∈S,

(11) $${\left({\hat{M}}_{n+1}\right)}_{xy}≔\frac{p_{N-n}\left(x\right)}{p_{N-n}\left(y\right)}{\left(M_{N-n}\right)}_{yx}$$

for 0≤n≤N−1. Notice that ${\hat{M}}_{n}$ is a stochastic matrix for 1≤n≤N, as we have for all y∈S

$$\begin{array}{cc} \sum_{x\in S}{\left({\hat{M}}_{n}\right)}_{xy} & =\sum_{x\in S}{\left(M_{N+1-n}\right)}_{yx}\frac{p_{N+1-n}\left(x\right)}{p_{N+1-n}\left(y\right)}\\ \; & =\frac{1}{p_{N+1-n}\left(y\right)}\sum_{x\in S}{\left(M_{N+1-n}\right)}_{yx}p_{N+1-n}\left(x\right)=1, \end{array}$$

where we applied (11) in the first equality and the fact that $p_{n}$ is a stationary distribution for $M_{n}$ for 1≤n≤N by the assumption in the last equality. Thus, Y is well defined. Note that, by definition we have

$$\begin{array}{cc} P(Y_{n+1}=x_{n+1}|Y_{n}=x_{n}) & =P(Y_{n+1}=x_{n+1}|Y_{n}=x_{n},..,Y_{0}=x_{0})\\ \; & ={\left({\hat{M}}_{n+1}\right)}_{x_{n+1}x_{n}} \end{array}$$

for 0≤n≤N−1, $\left(x_{0},..,x_{n+1}\right)\in S^{n+2}$. We can now use Y to show the result:

$$\begin{array}{cc} \langle e^{-\beta W(X)}\rangle & \overset{\left(i\right)}{=}\sum_{x_{0},..,x_{N}\in S}P\left(X_{0}=x_{0}\right){\left(M_{1}\right)}_{x_{1}x_{0}}{\left(M_{2}\right)}_{x_{2}x_{1}}\cdots {\left(M_{N}\right)}_{x_{N}x_{N-1}}\times \\ \; & \;\;\;\times \frac{e^{-\beta E_{1}\left(x_{0}\right)}}{e^{-\beta E_{0}\left(x_{0}\right)}}\cdots \frac{e^{-\beta E_{N}\left(x_{N-1}\right)}}{e^{-\beta E_{N-1}\left(x_{N-1}\right)}}\\ \; & \overset{\left(ii\right)}{=}\sum_{x_{0},\ldots ,x_{N}\in S}P\left(X_{0}=x_{0}\right){\left({\hat{M}}_{N}\right)}_{x_{0}x_{1}}\frac{e^{-\beta E_{1}\left(x_{1}\right)}}{e^{-\beta E_{1}\left(x_{0}\right)}}\cdots {\left({\hat{M}}_{1}\right)}_{x_{N-1}x_{N}}\times \\ \; & \;\;\;\times \frac{e^{-\beta E_{N}\left(x_{N}\right)}}{e^{-\beta E_{N}\left(x_{N-1}\right)}}\frac{e^{-\beta E_{1}\left(x_{0}\right)}}{e^{-\beta E_{0}\left(x_{0}\right)}}\cdots \frac{e^{-\beta E_{N}\left(x_{N-1}\right)}}{e^{-\beta E_{N-1}\left(x_{N-1}\right)}}\\ \; & \overset{\left(iii\right)}{=}\frac{Z_{N}}{Z_{0}}\sum_{x_{0},\ldots ,x_{N}\in S}P\left(Y_{0}=x_{N}\right){\left({\hat{M}}_{1}\right)}_{x_{N-1}x_{N}}\cdots {\left({\hat{M}}_{N}\right)}_{x_{0}x_{1}}\\ \; & \overset{\left(iv\right)}{=}e^{-\beta \Delta F}, \end{array}$$

where we use the Markov property in (i) and apply (11) in (ii). In (iii), we cancel the repeated terms coming from the definition of ${\left({\hat{M}}_{n}\right)}_{n=1}^{N}$ and from $e^{-\beta W(x)}$, since we have

(12) $$\begin{array}{ccc} \sum_{n=1}^{N}E_{n}\left(x_{n}\right)-E_{n}\left(x_{n-1}\right) & = & E_{N}\left(x_{N}\right)-E_{1}\left(x_{0}\right)+\sum_{n=1}^{N-1}E_{n}\left(x_{n}\right)-E_{n+1}\left(x_{n}\right)\\ & = & E_{N}\left(x_{N}\right)-E_{0}\left(x_{0}\right)-W\left(x\right) \end{array}$$

which leads to

$$\begin{array}{cc} \; & P\left(X_{0}=x_{0}\right)\frac{e^{-\beta E_{1}\left(x_{1}\right)}}{e^{-\beta E_{1}\left(x_{0}\right)}}..\frac{e^{-\beta E_{N}\left(x_{N}\right)}}{e^{-\beta E_{N}\left(x_{N-1}\right)}}\frac{e^{-\beta E_{1}\left(x_{0}\right)}}{e^{-\beta E_{0}\left(x_{0}\right)}}..\frac{e^{-\beta E_{N}\left(x_{N-1}\right)}}{e^{-\beta E_{N-1}\left(x_{N-1}\right)}}\\ \; & =\frac{e^{-\beta E_{0}\left(x_{0}\right)}}{Z_{0}}e^{-\beta (\sum_{n=1}^{N}E_{n}\left(x_{n}\right)-E_{n}\left(x_{n-1}\right))}e^{-\beta W(x)}\\ \; & =\frac{1}{Z_{0}}e^{-\beta (E_{0}\left(x_{0}\right)+E_{N}\left(x_{N}\right)-E_{0}\left(x_{0}\right)-W\left(x\right)+W\left(x\right))}\\ \; & =\frac{Z_{N}}{Z_{0}}P\left(Y_{0}=x_{N}\right). \end{array}$$

Lastly, we apply the definition of ΔF and the normalization of Y in (iv). □

The main purpose of proving Theorem 1 is to show that Jarzynski’s equality can be obtained under milder conditions than the ones that were considered before in decision-making. It should be noted that, in contrast to the usual approaches such as [8], where Y is assumed to be the time reversal of X, here, it is just a convenient mathematical object for the proof. A similar approach to Jarzynski’s equality in the context of continuous-time Markov chains can be found in [36]. Our approach to the discrete-time case in Theorem 1 is much simpler, since we do not need measure-theoretic concepts nor smoothness assumptions.

## 4. Crooks’ Fluctuation Theorem for Markov Chains

The original derivation of Crooks’ fluctuation theorem for Markovian dynamics [7,8,15] was carried out using a definition of work different from the one in (4). In this section, we derive the theorem for Markov chains using (4) and comment on the difference between these approaches in the discussion. As discussed in the Introduction, an additional hypothesis is needed for the result to hold in our setup. We derive Crooks’ fluctuation theorem using this additional assumption in Theorem 2 and Corollary 2 below, and then, in Proposition 3, we provide an example where this requirement is violated and the theorem is false everywhere.

Before proving the intermediate results and, finally, Crooks’ fluctuation theorem, let us briefly sketch the procedure we follow throughout this section. We start with Proposition 1, where we use the same Markov chain Y that we defined in the proof of Theorem 1 and obtain, along similar lines, a more precise relation between X and Y, in particular between the probability of some realization of X and that of the same realization (with the events taking place in reversed order) of Y. As a matter of fact, we show that, for a given X, Y is (roughly) the only Markov chain fulfilling such a relation. Then, in Proposition 2, we show how the driving signals of X and Y are related. In order to do so, we exploit the relation between the equilibrium distributions of X and Y, which comes from the fact the both Markov chains share the same equilibrium distributions. By combining these two propositions, we reach a relation between the probability distributions of the driving signals of X and Y in Theorem 2. Lastly, in Corollary 2, we impose an extra condition on the equilibrium distributions of X in order to obtain Crooks’ fluctuation theorem in its usual form. We proceed now to show the details involved in the argument we just presented. We start by proving Proposition 1.

**Proposition 1.**  *If $X={\left(X_{n}\right)}_{n=0}^{N}$ is a Markov chain on a finite state space S whose initial distribution $p_{0}$ has non-zero entries and whose transition matrices ${\left(M_{n}\right)}_{n=1}^{N}$ are irreducible, then there exists a unique Markov chain $Y={\left(Y_{n}\right)}_{n=0}^{N}$ such that $Y_{0}∼p_{N}$, ${\left({\hat{M}}_{n+1}\right)}_{xx}={\left(M_{N-n}\right)}_{xx}$∀x∈S, 0≤n≤N−1, where ${\left({\hat{M}}_{n}\right)}_{n=1}^{N}$ are the transition matrices of Y, and for $x=\left(x_{0},x_{1},\ldots ,x_{N}\right)\in S^{N+1}$,*

(13) $$\begin{array}{cc} \; & P(X_{1}=x_{1},\ldots ,X_{N}=x_{N}|X_{0}=x_{0})\\ \; & =P\left(Y_{1}=x_{n-1},\ldots ,Y_{N}=x_{0}|Y_{0}=x_{N}\right)\;e^{-\beta Q(x)} \end{array}$$

*for any family of energies $E={\left(E_{n}\right)}_{n=0}^{N}$ of X. Moreover, this unique Y satisfies*

(14) $$P\left(X=x\right)\;=\;P\left(Y=x^{R}\right)\;e^{\beta (W(x)-\Delta F)},$$

*where $W=W_{X,E}$, $\Delta F=\Delta F_{X,E}$, and $x^{R}≔\left(x_{N},\ldots ,x_{0}\right)$ denotes the reversal of x. In particular, the probability of X following x is the same as the probability of Y following $x^{R}$ if and only if ΔF=W(x).*

**Proof.**  Consider the Markov chain $Y={\left(Y_{n}\right)}_{n=0}^{N}$ defined in the proof of Theorem 1, which is well defined, since we have the same hypotheses. We can proceed in the same way as in Theorem 1 to obtain

$$\begin{array}{cc} \; & P\left(X_{1}=x_{1},\ldots ,X_{N}=x_{N}|X_{0}=x_{0}\right)\overset{\left(i\right)}{=}{\left(M_{1}\right)}_{x_{1}x_{0}}\cdots {\left(M_{N}\right)}_{x_{N}x_{N-1}}\\ \; & \overset{\left(ii\right)}{=}\frac{e^{-\beta E_{1}\left(x_{1}\right)}}{e^{-\beta E_{1}\left(x_{0}\right)}}\cdots \frac{e^{-\beta E_{N}\left(x_{N}\right)}}{e^{-\beta E_{N}\left(x_{N-1}\right)}}{\left({\hat{M}}_{N}\right)}_{x_{0}x_{1}}\cdots {\left({\hat{M}}_{1}\right)}_{x_{N-1}x_{N}}\\ & \overset{\left(iii\right)}{=}e^{-\beta Q(x)}P\left(Y_{1}=x_{n-1},\ldots ,Y_{N}=x_{0}|Y_{0}=x_{N}\right), \end{array}$$

where we applied the Markov property of X in (i), (11) in (ii), and the definition of *Q* plus the Markov property of Y in (iii). This proves the existence of a Markov chain with the desired properties. Moreover, we have

$$\begin{array}{cc} P(X=x) & \overset{\left(i\right)}{=}\frac{e^{-\beta E_{0}\left(x_{0}\right)}}{Z_{0}}e^{-\beta Q(x)}P\left(Y_{1}=x_{n-1},\ldots ,Y_{N}=x_{0}|Y_{0}=x_{N}\right)\\ \; & \overset{\left(ii\right)}{=}\frac{Z_{N}}{Z_{0}}e^{-\beta (Q\left(x\right)-\left(E_{N}\left(x_{N}\right)-E_{0}\left(x_{0}\right)\right))}\;P\left(Y_{0}=x_{N},\ldots ,Y_{N}=x_{0}\right)\\ \; & \overset{\left(iii\right)}{=}e^{\beta (W(x)-\Delta F)}\;P\left(Y=x^{R}\right), \end{array}$$

where we applied (4), the definition of conditional probability and the fact $X_{0}$ follows $p_{0}$ in (i), the definition of the conditional probability, the fact that $Y_{0}$ follows $p_{N}$ in (ii), and both the definitions of *Q* and ΔF plus (12) in (iii).

It remains to show the uniqueness of Y. Assume $Z={\left(Z_{n}\right)}_{n=0}^{N}$ is a Markov chain with transition matrices ${\left(M_{n}^{\prime }\right)}_{n=1}^{N}$ such that $Z_{0}∼p_{N}$, (13) holds, and ${\left(M_{n+1}^{\prime }\right)}_{xx}={\left(M_{N-n}\right)}_{xx}$ for all x∈S and 0≤n≤N−1. Consider some *n* such that 1<n<N and some a,b∈S with a≠b. By (13), we have

$$\begin{array}{cc} \; & P\left(X_{1}=a,\ldots ,X_{N-(n-2)}=a,X_{N-(n-1)}=b,\ldots ,X_{N}=b|X_{0}=a\right)e^{-\beta E_{N-(n-1)}\left(a\right)}\\ & =P\left(Z_{1}=b,\ldots ,Z_{n-1}=b,Z_{n}=a,\ldots ,Z_{N}=a|Z_{0}=b\right)e^{-\beta E_{N-(n-1)}\left(b\right)}. \end{array}$$

Applying the Markov property, the fact that ${\left(M_{n+1}^{\prime }\right)}_{xx}={\left(M_{N-n}\right)}_{xx}$∀x∈S, 0≤n≤N−1, and the definition of $E_{N-(n-1)}$, we obtain

$${\left(M_{n}^{\prime }\right)}_{ab}={\left(M_{N-(n-1)}\right)}_{ba}\frac{p_{N-(n-1)}\left(a\right)}{p_{N-(n-1)}\left(b\right)}={\left({\hat{M}}_{n}\right)}_{ab}.$$

Since the argument also works for n=1 and n=N, the case a=b holds by definition, and $Y_{0},Z_{0}∼p_{N}$, we have Z=Y. □

We say X is *microscopically reversible* [15] if (13) is satisfied for Y being the time reversal of X, i.e., if the unique Markov chain Y that exists by Proposition 1 has initial distribution $p_{N}$ and transition matrices ${\left(M_{N-n+1)}\right)}_{n=1}^{N}$. Notice, if this property holds, then (14) relates the probability of observing a realization x of a Markov chain X with that of observing the reversed realization $x^{R}$ in the time reversal Y of X, that is when starting with the equilibrium distribution of the last environment and choosing according to the same conditional probabilities, but in reversed order. This is the case if and only if the transition matrices of X satisfy detailed balance, as we show in the following lemma.

**Lemma 2.**  *If $X={\left(X_{n}\right)}_{n=0}^{N}$ is a Markov chain on a finite state space S with irreducible transition matrices ${\left(M_{n}\right)}_{n=1}^{N}$ and initial distribution with non-zero entries, then $M_{n}$ satisfies detailed balance for 1≤n≤N if and only if X is microscopically reversible.*

**Proof.**  If X satisfies detailed balance, that is if each $p_{n}$ satisfies (1), then by the definition of $\hat{M}$ in (11), we have ${\hat{M}}_{n}=M_{N-(n-1)}$ for each 1≤n≤N. Hence, in this case, the Markov chain Y constructed in Theorem 1 and Proposition 1 is the time reversal of X, and so, X is microscopically reversible by definition.

It remains to show that, if X is microscopically reversible, then its transition matrices satisfy detailed balance. Let Y be the time reversal of X. Since, by assumption, $Y_{0}∼p_{N}$ and ${\hat{M}}_{N-(n-1)}=M_{n}$, where ${\left({\hat{M}}_{n}\right)}_{n=1}^{N}$ are the transition matrices of Y, we can follow the proof of Proposition 1 to obtain for 1≤n≤N

$${\left(M_{n}\right)}_{ab}={\left({\hat{M}}_{N-(n-1)}\right)}_{ab}={\left(M_{n}\right)}_{ba}\frac{p_{n}\left(a\right)}{p_{n}\left(b\right)}.$$

Thus, for each 1≤n≤N, $p_{n}$ satisfies detailed balance with respect to $M_{n}$. □

In particular, this means that, if X satisfies detailed balance, then the time reversal Y of X satisfies (14), that is

(15) $$P\left(X=x\right)=P\left(Y=x^{R}\right)\;e^{\beta (W(x)-\Delta F)}=P\left(Y=x^{R}\right)\;e^{\beta W^{d}\left(x\right)}$$

for any family of energies $E={\left(E_{n}\right)}_{n=0}^{N}$ of X, where $W^{d}≔W_{X}^{d}$ is the dissipated work of X. Thus, in this case, dissipated work is an unambiguous measure of the discrepancy between the probability of observing a realization of X and the probability of observing the same trajectory in reversed order in the time reversal of X. We have, hence, an unambiguous measure of *hysteresis* (see Section 5).

Before showing Theorem 2 and Crooks’ fluctuation theorem, we relate the work of X with that of its time reversal.

**Proposition 2.**  *If $X={\left(X_{n}\right)}_{n=0}^{N}$ is a Markov chain on a finite state space S with the initial distribution with non-zero entries and irreducible transition matrices, then there exists a Markov chain $Y={\left(Y_{n}\right)}_{n=0}^{N}$ and a constant k∈R such that*

(16) $$W_{Y,\hat{E}}\left(x^{R}\right)=-W_{X,E}\left(x\right)+E_{1}\left(x_{0}\right)-E_{0}\left(x_{0}\right)+k\;\forall x\in S^{N+1},$$

*where E and $\hat{E}$ are families of energies of X and Y, respectively. Moreover, if the stationary distribution $p_{1}$ of $M_{1}$ coincides with the initial distribution $p_{0}$ of X, then there exists a constant k∈R such that*

(17) $$W_{Y,\hat{E}}\left(x^{R}\right)=-W_{X,E}\left(x\right)+k\;\forall x\in S^{N+1}.$$

**Proof.**  Let $Y={\left(Y_{n}\right)}_{n=0}^{N}$ be the Markov chain defined in the proof of Theorem 1. Since work is well defined (up to a constant) for both X and Y, by Lemma A2 (see Appendix A), we can use the relation between the energy functions of both chains in (A2) to show (16). We have

$$\begin{array}{cc} W_{Y,\hat{E}}\left(x^{R}\right) & =\sum_{n=0}^{N-1}{\hat{E}}_{n+1}\left(x_{n}^{R}\right)-{\hat{E}}_{n}\left(x_{n}^{R}\right)\\ \; & \overset{\left(i\right)}{=}\sum_{n=1}^{N-1}{\hat{E}}_{n+1}\left(x_{n}^{R}\right)-{\hat{E}}_{n}\left(x_{n}^{R}\right)+\hat{c}\\ \; & \overset{\left(ii\right)}{=}\sum_{n=1}^{N-1}E_{N-n}\left(x_{N-n}\right)-E_{N-(n-1)}\left(x_{N-n}\right)+k\\ \; & \overset{\left(iii\right)}{=}\sum_{m=1}^{N-1}E_{m}\left(x_{m}\right)-E_{m+1}\left(x_{m}\right)+k\\ \; & =-W_{X,E}\left(x\right)+E_{1}\left(x_{0}\right)-E_{0}\left(x_{0}\right)+k, \end{array}$$

where in (i) we defined $\hat{c}≔\hat{E_{1}}-\hat{E_{0}}$, which is a constant since ${\hat{E}}_{0}=E_{N}+k_{0}$ and ${\hat{E}}_{1}=E_{N}+k_{1}$ by Lemma A2 (see Appendix A). In (ii), we applied the definition of $x^{R}$ and (A2), cancelled the repeated $k_{n}$, defined as in Lemma A2, for all 1<n<N, and introduced $k≔k_{N}-k_{1}+\hat{c}=\left({\hat{E}}_{N}-E_{1}\right)-\left({\hat{E}}_{1}-E_{N}\right)+\left({\hat{E}}_{1}-{\hat{E}}_{0}\right)=\left({\hat{E}}_{N}-E_{1}\right)-\left({\hat{E}}_{0}-E_{N}\right)$. In (iii), we rewrite the sum in terms of m≔N−n.

For the second statement, notice that, if $p_{0}$ is the stationary distribution of $M_{1}$, then there exists a constant *c* such that $E_{1}=E_{0}+c$ by (2). Thus, we have $k≔\left({\hat{E}}_{N}-E_{0}\right)-\left({\hat{E}}_{0}-E_{N}\right)=\left(E_{1}-E_{0}\right)+\left({\hat{E}}_{N}-E_{1}\right)-\left({\hat{E}}_{0}-E_{N}\right)=c+k^{\prime }=E_{1}\left(x_{0}\right)-E_{0}\left(x_{0}\right)+k^{\prime }$ for all $x_{0}\in S$, where $k^{\prime }$ is the constant in (16). □

If detailed balance holds, then (16) and (17) relate the work along a realization x of X with the reversed realization $x^{R}$ of its time reversal Y. More precisely, we obtain the following corollary.

**Corollary 1** (When work is odd under time reversal)**.**  *If all transition matrices of X in Proposition 2 satisfy detailed balance and Y is the time reversal of X, then the constants k in (16) and (17) can be taken to be zero.*

**Proof.**  For the constant in (16), we simply choose ${\hat{E}}_{N}=E_{1}$ and ${\hat{E}}_{0}=E_{N}$, and for the constant in (17), we choose ${\hat{E}}_{N}=E_{0}$ and ${\hat{E}}_{0}=E_{N}$, which we can do since $p_{0}$ is the stationary distribution of $M_{1}$ and, by Lemma A2 (see Appendix A), also that of ${\hat{M}}_{N}$. □

**Remark 1.**  *Note that choosing the energy functions in Corollary 1 is unnecessary whenever both X and Y are thermodynamic processes. Although energy is defined only up to a constant in thermodynamics, it would make no sense to pick the constants differently when dealing with a system where the same dynamics occur more than once. Thus, there, we have ${\hat{E}}_{n}=E_{N-(n-1)}$ for 1≤n≤N, ${\hat{E}}_{0}={\hat{E}}_{1}$, and in case $p_{0}$ is a stationary distribution of $M_{1}$, $E_{1}=E_{0}={\hat{E}}_{N}$. In particular, when taking Y to be the time reversal of X in thermodynamics, we always have k=0 in (16), and in case $p_{0}$ is a stationary distribution of $M_{1}$, k=0 in (17).*

**Figure 2:**
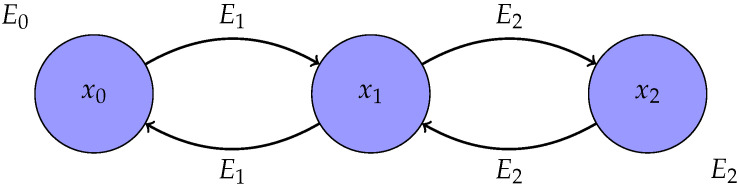
Simple example showing how the asymmetry between X and its time reversal Y manifests in thermodynamics. We consider $x=(x_{0},x_{1},x_{2})$ a trajectory, $\left(E_{0},E_{1},E_{2}\right)$ with $E_{1}\left(x_{0}\right)\neq E_{0}\left(x_{0}\right)$ the energy functions of X (upper line of arrows), and by Remark 1, $\left(E_{2},E_{2},E_{1}\right)$ the energy functions of Y (bottom line of arrows). We have $W_{X}\left(x\right)=E_{2}\left(x_{1}\right)-E_{1}\left(x_{1}\right)+E_{1}\left(x_{0}\right)-E_{0}\left(x_{0}\right)$ and $W_{Y}\left(x^{R}\right)=E_{1}\left(x_{1}\right)-E_{2}\left(x_{1}\right)$. As a result, $W_{Y}\left(x^{R}\right)=-W_{X}\left(x\right)+E_{1}\left(x_{0}\right)-E_{0}\left(x_{0}\right)$, in accordance with (16) and Corollary 1. Thus, thermodynamic work is *not* odd under time reversal in general.

The non-constant term $E_{1}\left(x_{0}\right)-E_{0}\left(x_{0}\right)$ in (16), which remains even when X satisfies detailed balance, follows from an asymmetry between X and Y. In particular, X goes from $E_{0}$ to $E_{N}$, whereas Y goes from $E_{N}$ to $E_{1}$, because while $X_{0}∼p_{0}\propto e^{-\beta E_{0}}$ and the stationary distribution of $M_{N}$ is $p_{N}\propto e^{-\beta E_{N}}$, which is also the initial distribution of Y, the stationary distribution of the final transition matrix ${\hat{M}}_{N}$ of Y is $p_{1}\propto e^{-\beta E_{1}}$ (and not $p_{0}$). Furthermore, while X may begin with a change in the energy function, since $p_{1}\neq p_{0}$ is allowed, Y does not, as $p_{N}$ is both the initial distribution of Y and the stationary distribution of ${\hat{M}}_{1}$. An example can be found in Figure 2. This asymmetry is erased if we assume that $p_{0}$ is the stationary distribution of $M_{1}$, in which case, for Markov chains X that satisfy detailed balance, the work along any realization of X has the opposite sign of the work along the reversed realization of Y. That is, thermodynamic work becomes *odd under time reversal*.

The following theorem contains Crooks’ fluctuation theorem as the special case when X satisfies detailed balance (see Corollary 2 below).

**Theorem 2.**  *If $X={\left(X_{n}\right)}_{n=0}^{N}$ is a Markov chain on a finite state space S whose initial distribution $p_{0}$ has non-zero entries, whose transition matrices ${\left(M_{n}\right)}_{n=1}^{N}$ are irreducible, and where $p_{0}$ is the stationary distribution of $M_{1}$, that is $p_{1}=p_{0}$, then there exists a Markov chain $Y={\left(Y_{n}\right)}_{n=0}^{N}$ and a constant k∈R such that*

(18) $$\frac{P(W_{X,E}=w)}{P(W_{Y,\hat{E}}=-w+k)}=e^{\beta (w-\Delta F)}\;\forall w\in \mathrm{supp}(P\left(W_{X,E}\right)),$$

*where $E={\left(E_{n}\right)}_{n=0}^{N}$ and $\hat{E}={\left({\hat{E}}_{n}\right)}_{n=0}^{N}$ are families of energies of X and Y, respectively, and $\mathrm{supp}(P\left(W_{X,E}\right))$ denotes the support of the probability distribution of $W_{X,E}$, that is the values that can be taken by $W_{X,E}$ with non-zero probability.*

**Proof.**  Let $Y={\left(Y_{n}\right)}_{n=0}^{N}$ be the Markov chain defined in the proof of Theorem 1. Note that, for any family of energies E of X, there exists a constant *c* such that $E_{1}=E_{0}+c$ by (2), since $p_{0}$ is the stationary distribution of $M_{1}$. Given some $w\in W_{X,E}\left(S^{N+1}\right)$, we have

$$\begin{array}{cc} P(W_{X,E}=w) & =\sum_{x\in W_{X,E}^{-1}\left(w\right)}P\left(\left(X_{0},..,X_{N}\right)=x\right)\\ \; & \overset{\left(i\right)}{=}e^{\beta (w-\Delta F)}\sum_{x\in W_{X,E}^{-1}\left(w\right)}P\left(\left(Y_{0},..,Y_{N}\right)=x^{R}\right)\\ \; & \overset{\left(ii\right)}{=}e^{\beta (w-\Delta F)}\sum_{x\in S^{N+1}:\;W_{Y,\hat{E}}\left(x^{R}\right)=-w+k}P\left(\left(Y_{0},..,Y_{N}\right)=x^{R}\right)\\ \; & =e^{\beta (w-\Delta F)}\;P\left(W_{Y,\hat{E}}=-w+k\right), \end{array}$$

where we applied (14) in (i) and Proposition 2 plus the fact that $E_{1}=E_{0}+c$ in (ii). To obtain (18), it remains to show that $P(W_{Y,\hat{E}}=-w+k)>0$ for all $w\in \mathrm{supp}(P\left(W_{X,E}\right))$. By definition, there exists some $x\in S^{N+1}$ such that $W_{X,E}\left(x\right)=w$ and P(X=x)>0. By Proposition 2, $W_{Y,\hat{E}}\left(x^{R}\right)=-w+k$. Since P(X=x)>0 and the entries of the (unique) stationary distributions of X are also non-zero by Lemma 1, we can use (11) plus the Markov property for both X and Y to show $P(Y=x^{R})>0$, implying $P(W_{Y,\hat{E}}=-w+k)>0$. □

As the special case when each transition matrix of X in Theorem 2 satisfies detailed balance, we obtain Crooks’ fluctuation theorem modified by the additional assumption of $p_{0}=p_{1}$.

**Corollary 2** (Crooks’ fluctuation theorem for Markov chains)**.**  *If all transition matrices ${\left(M_{n}\right)}_{n=1}^{N}$ of the Markov chain $X={\left(X_{n}\right)}_{n=0}^{N}$ in Theorem 2 satisfy detailed balance, then (18) holds with k=0 and Y being the time reversal of X, that is the Markov chain with initial distribution $p_{N}$ and transition matrices ${\left(M_{N-(n-1)}\right)}_{n=1}^{N}$.*

**Proof.**  As can be seen from the proof of Theorem 2 and Corollary 1, in the case of detailed balance, we can choose Y to be the time reversal of X. Moreover, the origin of the constant *k* in Theorem 2 is Equation (17). By Corollary 1, this constant can be set to zero if the transition matrices of X satisfy detailed balance. □

Notice, in most of the literature on Crooks’ fluctuation theorem, one writes $P^{F}\left(W\right)$ for the probability $P(W_{X,E})$ of the work along the so-called *forward process* X and $P^{B}\left(W\right)$ for the probability $P(W_{Y,\hat{E}})$ of the work along the so-called *backward process* Y (the time reversal of X), so that, by Corollary 2, under detailed balance, Equation (18) reads

(19) $$\frac{P^{F}\left(W=w\right)}{P^{B}\left(W=-w\right)}=e^{\beta (w-\Delta F)}\;.$$

The condition that $p_{0}$ is the stationary distribution of $M_{1}$ is not necessary in Crooks’ original work [7,8,15,17]. Nonetheless, it is fundamental in our approach: Crooks’ fluctuation theorem can even be false for every work value if $p_{0}$ is not the stationary distribution of $M_{1}$, as we show in Proposition 3.

**Proposition 3.**  *If $p_{0}$ is not the stationary distribution of $M_{1}$, then there exist Markov chains where Crooks’ fluctuation theorem is false everywhere, despite the other assumptions in Theorem 2 and Corollary 2 being fulfilled.*

**Proof.**  We consider a state space with three components S={A,B,C}, a Markov chain with two steps $X=(X_{0},X_{1})$ and a,b,c>0, where b<c and a+b+c=1. We take $E_{0}$ as the energy function associated with $X_{0}$, where $E_{0}\left(A\right)=\log\frac{1}{a}$, $E_{0}\left(B\right)=\log\frac{1}{b}$ and $E_{0}\left(C\right)=\log\frac{1}{c}$, and $E_{1}$ as the energy function associated with $X_{1}$, where $E_{1}\left(A\right)=E_{0}\left(A\right)$, $E_{1}\left(B\right)=E_{0}\left(C\right)$ and $E_{1}\left(C\right)=E_{0}\left(B\right)$. Taking β=1, we obtain both free energies $F_{1}$ and $F_{0}$ equal to one. Notice that we have $p_{0}=\left(a,b,c\right)$, with non-zero entries, and $p_{1}=\left(a,c,b\right)$. Notice, also, that $W_{X}\left(S^{2}\right)=\left\{w_{A},w_{B},w_{C}\right\}$, where $w_{C}≔E_{1}\left(C\right)-E_{0}\left(C\right)>0$, $w_{B}≔E_{1}\left(B\right)-E_{0}\left(B\right)<0$, and $w_{A}≔E_{1}\left(A\right)-E_{0}\left(A\right)=0$. We fix ${\hat{E}}_{1}={\hat{E}}_{0}=E_{1}$. We can easily see that (19) is not defined for $w=w_{B},w_{C}$ and that it is false, although defined, for $w=w_{A}$. We have $W_{Y}\left(x\right)=0$$\forall x\in S^{2}$, since $p_{1}$ is both the starting distribution of the time reversal of X and the stationary distribution of its only transition matrix. Thus, we have $P^{F}\left(W=w_{C}\right)=P\left(X_{0}=C\right)=c>0$ and $P^{B}\left(W=-w_{C}\right)=0$, which means (19) is not defined at $W=w_{C}$. We obtain, analogously, that it is not defined for $W=w_{B}$. For $W=w_{A}$, we have $P^{B}\left(W=-w_{A}\right)=1$ and

$$P^{F}\left(W=w_{A}\right)=P\left(X_{0}=A\right)=a<1=e^{\beta w_{A}}$$

which means (19) is defined and false there. Although the argument is independent of the transition matrix for X, fix

$$M_{1}≔\left(\begin{array}{ccc} a & a & a\\ c & c & c\\ b & b & b \end{array}\right)$$

for completeness, since it has non-zero entries and fulfils detailed balance with respect to $p_{1}$. □

## 5. Discussion: Application to Decision-Making

The bridge connecting thermodynamics and decision-making is an optimization principle directly inspired by the *maximum entropy principle* [27,30,37,38]. In particular, given a finite set of possible choices *S*, the optimal behaviour is given by the distribution $p\in P_{S}$ that optimally trades utility and uncertainty according to the following optimization principle:

(20) $$p=\arg\max_{q\in P_{S}}\left\{H\left(q\right)|E_{q}\left[U\right]\geq U_{0}\right\},$$

where $P_{S}$ is the set of probability distributions over *S*, *H* is the Shannon entropy, $E_{q}\left[\;\cdot \;\right]$ denotes the expected value over $q\in P_{S}$, and U:S→R is a *utility* function, that is a function that assigns larger values to options in *S* that are preferred by the decision-maker. Note that the main difference between (20) and the maximum entropy principle is the the substitution of an energy function E:S→R by a utility *U*, which behaves as a negative energy function U=−E (in the sense that it is the *force* that opposes uncertainty). As a result of their similarity, both principles yield the same result, namely the Boltzmann distribution:

$$p\left(x\right)=\frac{1}{Z}e^{\beta U(x)}=\frac{1}{Z}e^{-\beta E(x)}\;\forall x\in S,$$

where β is a trade-off parameter between uncertainty and utility/energy and *Z* is a normalization constant.

The analogy between thermodynamics and decision-making can be taken further by considering not only their optimal distribution, but how they transition between different distributions in the path towards the optimal one. Here, the notion of uncertainty is useful again, although this time, it is relative to the optimal distribution *p*. More specifically, we can think of the transitions the decision-maker undergoes as being driven by the reduction of uncertainty with respect to the optimal distribution *p*, which can be modelled by the dual of *p*-majorization [39]. In this approach, given $q,q^{\prime }\in P_{S}$, the decision-maker transitions from *q* to $q^{\prime }$, which we denote by $q{⪯}_{p}q^{\prime }$, if $q^{\prime }$ is *closer* to *p* than *q* (see [39] for a rigorous definition of the dual of ${⪯}_{p}$ and, hence, of *closer*). For us, the important fact about ${⪯}_{p}$ is that, as it turns out ([40], Theorem 2), the transitions that are allowed by ${⪯}_{p}$ are precisely the ones that result from applying a transition matrix that has *p* as a stationary distribution. More precisely,

(21) $$q{⪯}_{p}q^{\prime }\Leftrightarrow q^{\prime }=M_{p}q,$$

where $M_{p}$ is a matrix whose rows are normalized and fulfils $M_{p}p=p$, that is a *stochastic* matrix for which $p\in P_{S}$ is a stationary distribution [33]. Importantly, the transition matrix assumption is common in the study of both thermodynamic and decision-making systems [8,15].

In our current study, the situation we have in mind is that of a decision-maker that has to make a sequence of decisions under varying environmental conditions. We model this as a stochastic process that behaves like a Markov chain and introduce, starting from a Markov chain, the thermodynamic tools we use to describe it: energy, partition function, free energy, work, heat, and dissipated work. The behaviour of the decision-maker then corresponds to a decision vector $\left(x_{0},x_{1},\ldots ,x_{n}\right)$ collected over *n* potentially different environments. In this most general decision-making scenario, where the environment is changing over time, the optimal distribution *p* is changing as well. Thus, we can regard the decision-making process as a sequence of transition matrices $M_{p}$, where each $M_{p}$ corresponds to a particular environment with optimal response *p*. We could imagine, for example, a gradient descent learner that would converge to *p* for any given environment, presuming we allow for sufficient gradient update steps. Otherwise, the gradient learner, or any other optimization-based decision-making agent (e.g., following a Metropolis–Hastings optimization scheme), would lag behind the environmental changes and the environment would outpace the learner. In this general decision-making scenario, we can then study the relation between the optimal behaviour and the non-optimal one by fluctuation theorems [1,2,3] like Jarzynski’s equality [4,5,6] and Crooks’ fluctuation theorem [7,8]. While we focus on these two fluctuation theorems in our current study, similar arguments may be suitable to transfer other fluctuation theorems that have been considered in the thermodynamic literature (see, for example, [1]) to a decision-making scenario.

Jarzynski’s equality in decision-making:

Although the strong requirements in Lemma 2 were used in [8] to derive Jarzynski’s equality through (15) and were assumed in the only approach we know for it in decision-making [32], weaker assumptions that do not involve the time reversal of X are sufficient (cf. Theorem 1). The same properties have been used to derive Jarzynski’s equality following a different method in [4].

Example application: Jarzynski’s theorem:

In a decision scenario, the energy becomes a loss function that a decision-maker is trying to minimize. If this loss function changes over time, we can conceptually distinguish changes in loss that are induced externally by changes in the environment (e.g., given data), from changes in loss due to internal adaptation when a learning system changes its parameter settings. The externally induced changes in loss correspond to the physical concept of work and drive the adaptation process. Hence, we can consider the decision-theoretic equivalent of physical work as a driving signal: the (negative) surprise experienced by the decision-maker, given that it adds the (negative) surprise that he/she experiences at each step (which can be quantified by the difference in energy/utility evaluated at the decision-maker’s state when the environment changes) [32,41]. With this in mind, we can use Jarzynski’s equality to obtain a bound on the decision-maker’s expected surprise. In particular, by applying Jensens’ inequality on Jarzynski’s equality, one obtains

(22) $$\Delta F\leq E\left[W(X)\right].$$

(Note that this is a version of the second law of thermodynamics [2].) Hence, (22) provides a bound on the expected surprise. While a similar bound has been previously pointed out for decision-making systems [32], here, we re-derive it under a novel energy protocol and with weaker assumptions regarding time reversibility.

Crooks’ theorem in decision-making:

Even though the assumption that $p_{0}$ must be the stationary distribution of $M_{1}$ seems to restrict the applicability of Crooks’ fluctuation theorem in our decision-theoretic setup when compared to the usual thermodynamics one (see Corollary 2 and Proposition 3), it is actually not an issue from an experimental point of view when the Markov chains correspond to thermodynamic processes. This is the case because of the way one is able to sample from a Boltzmann distribution given a thermodynamic system. One of the assumptions in Corollary 2 is that $X_{0}$ should follow such a distribution for $E_{0}$. For this to be fulfilled, one needs to wait until the system relaxes to such a state. Because of that, one can think of any trajectory as having an additional point that was also sampled from the Boltzmann distribution for $E_{0}$. Thus, the assumption that $p_{0}$ is the equilibrium distribution of $M_{1}$ is always fulfilled, and the experimental range of validity of Crooks’ fluctuation theorem in our setup remains equal to the one in nonequilibrium thermodynamics [8,15]. In particular, the new constraint is fulfilled in previous experimental setups supporting the theorem (see, for example, [9] or [11]).

Example application: Crooks’ theorem:

Hysteresis is a well-known effect that takes place in some physical systems and refers to the difference in the system’s behaviour when interacting with a series of environments compared to its response when facing the same conditions in reversed order [2]. The same idea also applies to decision-making systems. In fact, hysteresis has been reported in both simulations of decision-making systems [32], as well as in biological decision-makers recorded experimentally [41,42]. Given that it refers to the difference between decisions when the order in which the environments are presented is reversed, (15) and (19) constitute quantitative measures of hysteresis. In particular, Reference [41] used this measure successfully to quantify hysteresis in human sensorimotor adaptation, where human learners had to solve a simple motor coordination task in a dynamic environment with changing visuomotor mappings. While a simple Markov model proved adequate to model sensorimotor adaptation, it should be noted that more complex learning scenarios involving long-term memory and abstraction would not be captured by such a simple model.

Detailed balance:

Detailed balance is not required neither for Jarzynski’s equality (Theorem 1), nor for the more general form of Crooks’ fluctuation theorem we presented in Theorem 2. It is, however, required in order to choose Y to be the time reversal of X, which leads to Crooks’ fluctuation theorem in Corollary 2.

While the definition of detailed balance (1) we adopted here is standard in the Markov chain literature, there is some ambiguity regarding its use in thermodynamics, where it has, at least, two more meanings. It is used both for the weaker condition that the Boltzmann distribution $p_{n}$ is a stationary distribution of $M_{n}$ for 1≤n≤N [4] and as a synonym of microscopic reversibility [8,15]. Although we have shown microscopic reversibility and detailed balance are indeed equivalent under some conditions (see Lemma 2), we followed its definition in [15], which is not the only one in the literature (see [35,43,44]).

Notice what is called a stationary distribution in the literature on Markov chains is referred to as a *nonequilibrium steady state* in thermodynamics [45]. In order for it to be an *equilibrium state*, it needs to fulfil detailed balance (1) with respect to the the transition matrix in question. Notice, also, detailed balance is not fulfilled in several applications of nonequilibrium thermodynamics throughout physics [46] and biology [47].

Continuous-time Markov chains:

Notice, in the case that we have a continuous-time Markov chain, work becomes an integral, where the integrand for X at x and the one for Y at $x^{R}$ differ, aside from the sign, in a single point. Thus, work is odd under time reversal and the assumption that $p_{0}=p_{1}$ can be dropped in both Theorem 2 and Corollary 2. However, the technical tools required to show Crooks’ fluctuation theorem or Jarzynski’s equality are technically more involved in the continuous-time case, as one can see in [36].

Continuous state space:

In case the state space is continuous, the results can be derived in a similar fashion. What we ought to notice is that, in this scenario, the role of the transition matrices is played by the densities of the Markov kernels (see for example [48]). These densities allow us to write conditions such as detailed balance analogously to how we do in the discrete case. In the case of Jarzynski’s equality for a Markov chain on a continuous state space X, one can see that the result follows like the one with a discrete state space. To convince ourselves, the only thing to take into account is the substitution of the sum in the expected value by the integral and that of the probability distribution by the density of the Markov kernel. Then, following the proof of Theorem 1, we can define a stochastic process Y whose density Markov kernels are defined through the stationary distributions and density Markov kernels of X, in analogy to how we defined them in Theorem 1. The rest follow exactly in the same fashion. Crooks’ fluctuation theorem requires a longer explanation, but, essentially, follows from the same considerations.

## 6. Conclusions

In this paper, we investigated the potential of thermodynamic fluctuation theorems to serve probabilistic laws of decision-making. In particular, we derived two thermodynamic fluctuation theorems, Jarzynski’s equality and Crooks’ theorem, in the context of general Markov chains X. We started by defining several thermodynamic concepts for Markov chains and discussing how these definitions do not correspond in general to the ones used in thermodynamics. Right after that, we derived Jarzynski’s equality in Theorem 1 without any assumption involving the time reversal of X. Thus, we improved on the previous attempt to derive it in the context of decision-making [32], which was based on the physical conventions pioneered in [8]. Regarding Crooks’ fluctuation theorem, we showed in Theorem 2, Corollary 2, and Proposition 3 that, in our decision-theoretic setup, it requires the additional assumption that the initial distribution of X must be the stationary distribution of its first transition matrix. This results from the fact that our notion of work is inherent to Markov chains, which contrasts with the definition used in previous derivations [8,15,17], where the work along the forward and backward paths was calculated in a different way for physical reasons. Instead, we calculated the work along the two paths in the same way, in order for the quantities involved in the final result to have an interpretation that is relevant for decision-making.

## Data Availability

Not applicable.

## Abbreviations

The following abbreviations are used in this manuscript:

MDPI	Multidisciplinary Digital Publishing Institute
DOAJ	Directory of open access journals
TLA	Three letter acronym
LD	Linear dichroism

## Appendix A

Here, we show two results relating a Markov chain X to the Markov chain Y defined by X as in Theorem 1. First, in Lemma A1, we relate the irreducibility of transition matrices for X with that of Y.

**Lemma A1.** 
*If $X={\left(X_{n}\right)}_{n=0}^{N}$ is a Markov chain on a finite state space S whose transition matrices are irreducible, then the transition matrices of the Markov chain $Y={\left(Y_{n}\right)}_{n=0}^{N}$ defined in Theorem 1 are irreducible.*

**Proof.** We denote by ${\left(M_{n}\right)}_{n=1}^{N}$ the transition matrices of X and by ${\left({\hat{M}}_{n}\right)}_{n=1}^{N}$ the ones of Y. Consider x,y∈S and ${\hat{M}}_{n}$ for some 1≤n≤N. Since $M_{N+1-n}$ is irreducible, there exist m∈N and $x_{0},x_{1},..,x_{m}$ where $x_{0}=y$ and $x_{m}=x$ such that

(A1) $$P\left(X_{m}=x_{m},..,X_{1}=x_{1}|X_{0}=x_{0}\right)={\left(M_{N+1-n}\right)}_{x_{m},x_{m-1}}..{\left(M_{N+1-n}\right)}_{x_{1},x_{0}}>0.$$

Hence, we have

$$\begin{array}{cc} \; & P\left(Y_{m}=x_{0},..,Y_{1}=x_{m-1}|Y_{0}=x_{m}\right)\overset{\left(i\right)}{=}{\left({\hat{M}}_{n}\right)}_{x_{0},x_{1}}..{\left({\hat{M}}_{n}\right)}_{x_{m-1},x_{m}}\\ \; & \overset{\left(ii\right)}{=}{\left(M_{N+1-n}\right)}_{x_{m},x_{m-1}}..{\left(M_{N+1-n}\right)}_{x_{1},x_{0}}\frac{p_{N+1-n}\left(x_{m-1}\right)}{p_{N+1-n}\left(x_{m}\right)}..\frac{p_{N+1-n}\left(x_{0}\right)}{p_{N+1-n}\left(x_{1}\right)}\\ \; & \overset{\left(iii\right)}{>}0 \end{array}$$

where we applied the Markov property of Y in (i), (11) in (ii), and (A1) plus the fact that, as $M_{N+1-n}$ is irreducible, $p_{N+1-n}\left(x\right)>0$∀x∈S by Lemma 1 in (iii). □

The connection between the irreducibility of the transition matrices of X and Y in Lemma A1 results in a relation between their energy functions, which we prove in Lemma A2.

**Lemma A2.** 
*If $X={\left(X_{n}\right)}_{n=0}^{N}$ is a Markov chain on a finite state space S whose initial distribution $p_{0}$ has non-zero entries and whose transition matrices ${\left(M_{n}\right)}_{n=1}^{N}$ are irreducible, $Y={\left(Y_{n}\right)}_{n=0}^{N}$ is the Markov chain defined in Theorem 1, $E={\left(E_{n}\right)}_{n=0}^{N}$ is a family of energy functions of X, and $\hat{E}={\left({\hat{E}}_{n}\right)}_{n=0}^{N}$ is a family of energy functions of Y, then there exist constants ${\left(k_{n}\right)}_{n=0}^{N}$ such that ${\hat{E}}_{0}=E_{N}+k_{0}$ and*

(A2)
$${\hat{E}}_{n+1}=E_{N-n}+k_{n+1}$$

*for 0≤n≤N−1.*

**Proof.** Since all transition matrices of X are irreducible and the same holds for Y by Lemma A1, we can apply Lemma 1 to Y and obtain that each of its transition matrices has a unique stationary distribution composed of non-zero entries. Thus, energy is well defined, up to a constant, for Y. Since $Y_{0}$ follows $p_{N}$ by definition, we have automatically there exists a constant $k_{0}$ such that ${\hat{E}}_{0}=E_{N}+k_{0}$. To obtain (A2), notice that we have ${\hat{M}}_{n+1}p_{N-n}=p_{N-n}$ for 0≤n≤N−1 by the definition of ${\left({\hat{M}}_{n}\right)}_{n=1}^{N}$:

(A3) $$\begin{array}{cc} \left({\hat{M}}_{n+1}p_{N-n}\right)\left(y\right) & =\sum_{x\in S}{\left({\hat{M}}_{n+1}\right)}_{yx}p_{N-n}\left(x\right)=\sum_{x\in S}{\left(M_{N-n}\right)}_{xy}p_{N-n}\left(y\right)\\ & =p_{N-n}\left(y\right), \end{array}$$

where we applied (11) in the second equality and the fact that $M_{N-n}$ is a stochastic matrix in the third. Thus, for 0≤n≤N−1, $p_{N-n}$ is the unique stationary distribution of ${\hat{M}}_{n+1}$, and there exists a constant $k_{n+1}$ such that ${\hat{E}}_{n+1}=E_{N-n}+k_{n+1}$. □
